# *Commiphora leptophloeos* Phytochemical and Antimicrobial Characterization

**DOI:** 10.3389/fmicb.2017.00052

**Published:** 2017-01-24

**Authors:** Jorge J. de Souza Pereira, Aline de P. C. Pereira, Jannyson J. B. Jandú, Josinete A. da Paz, Sergio Crovella, Maria T. dos Santos Correia, Jaqueline de Azevêdo Silva

**Affiliations:** ^1^Department of Genetics, Federal University of PernambucoRecife, Brazil; ^2^Laboratory of Immunopathology Keizo Asami, Federal University of PernambucoRecife, Brazil; ^3^Laboratory of Glycoproteins, Department of Biochemistry, Federal University of PernambucoRecife, Brazil; ^4^Department of Fundamental Chemistry, Federal University of PernambucoRecife, Brazil

**Keywords:** plant-derived products, drug discovery, antibacterial agents, multidrug resistance, hinokinin, *Mycobacterium tuberculosis*, *S. aureus*

## Abstract

*Commiphora leptophloeos* is a plant specie usually known for its medicinal purposes in local communities in Northeast Brazil. In order to evaluate its therapeutic potential, we aimed to determine the phytochemical and antimicrobial properties of *C. leptophloeos* extracts. Thin Layer Chromatography (TLC) was able to detect the presence of phenolic compounds, flavonoids and reducing sugars. Three phenolic compounds were identified by HPLC and described as Gallic, Chlorogenic and Protocatechuic acids. On the other hand, H^1^NMR analysis revealed the presence of hinokinin, a bioactive lignan further characterized in the present work. The minimum inhibitory concentration (MIC) values for hinokinin ranged from 0.0485 to 3.125 mg/mL in different *S. aureus* clinical isolates and showed a bactericidal activity against MRSA isolated from blood (MMC 0.40 mg/mL) and postoperative secretion (MMC = 3.125 mg/mL). *C. leptophloeos* extracts also showed antimicrobial activity against Mycobacterium species such as *M. smegmatis* (MIC = 12.5 mg/mL) and *M. tuberculosis* (MIC = 52 mg/mL). Additionally, we determined the toxicity of *C. leptophloeos* by *in vitro* HC_50_ tests with hemolytic activity detected of 313 ± 0.5 μg/mL. Our results showed that *C. leptophloeos* possesses inhibitory properties against MRSA as well as several other clinically important microorganisms. Furthermore, the present work is the first report of the presence of hinokinin in Commiphora genus.

## Introduction

The discovery of antibacterial agents was a breakthrough event in medicine and represented a landmark in human health. However, the widespread misuse of these agents has brought to light two major problems involving the treatment of bacterial infections: (i) the emergence of Multidrug-resistance Organisms (MDROs) and (ii) the existence of bacteria innately resistant to most antibiotics. The rising number of MDROs is an imminent threat worldwide, especially the Methicillin-resistant *Staphylococcus aureus* (MRSA), which has acquired a gene involved in the resistance to all available β-lactam antibiotics. In this scenario, Tuberculosis (TB), a disease caused by *Mycobacterium tuberculosis*, has co-evolved along with humans and remains as a public health issue to date. In 2015, TB was regarded as the world’s deadliest infectious disease (WHO, 2015), as 1 million children under 14 years old fell ill, 140.000 children died and over 53 million otherwise healthy children carried the TB bacillus. Moreover, TB is the leading cause of deaths by HIV-positive individuals as, in 2015, 1 in 3 HIV deceases was linked to TB infection. In the context of multidrug-resistance, approximately 480 000 people developed multidrug-resistant TB (MDR-TB) worldwide and the major causes are inappropriate treatment, misuse of drugs or use of poor quality medicines (Venturini et al., 2014; WHO, 2015).

The natural products found in medicinal plants are a promising source for new antibacterial compounds (Lawn et al., 2013; Zumla et al., 2013). Plant-derived antimicrobial compounds belong to an exceptionally wide diversity of classes, including terpenoids (Bhalodia et al., 2011), lignans (Teponno et al., 2016), alkaloids and peptides (Bhatter et al., 2016), phenolic compounds (Heleno et al., 2015) and coumarins (Bhatter et al., 2015, 2016). All of the aforementioned compounds are regarded as secondary metabolism products in plants, not strictly required for their survival, but usually conferring a positive effect for its use as medicinal purposes (Harborne, 1997; Kroymann, 2011).

*Commiphora leptophloeos*, usually known as Imburana of Sertão, belongs to the *Burseraceae* family, which includes trees and shrubs from tropical and subtropical regions, and is traditionally used by indigenous tribes as an infusion, tea or syrup for the treatment of their illness, such as infectious and inflammatory ones (Bennett and Prance, 2000; Silva et al., 2011). The Commiphora genus comprises over 150 species most of which are confined to Eastern Africa and are usually applied in traditional medicine (Abdel-Daim et al., 2015). In Brazil, it is found where the vegetation is exposed to adverse climate and soil conditions, typical of the Sertão physiognomy, a semi-arid region in Northeast Brazil characterized by a very dry and extremely hot weather throughout the year with low rainfall rates (Peña-Claros et al., 2012).

Therefore, plant species from Caatinga ecosystems, can become promising targets in the searches for new active substances. The aim of the present study included characterization of *C. leptophloeos* extracts, isolation of biomolecules and fractions with antimicrobial activity, and analysis of possible toxic effect in human blood cells.

## Materials and Methods

### Biological Material (Plant)

The stem bark of *C. leptophloeos* was collected at *Parque Nacional do Catimbau*, Pernambuco – Brazil. The authors confirm that the named authority *Instituto Chico Mendes de Conservação da Biodiversidade* granted permission (SISBIO 16806) for our described field searches. The botanical identification and the deposition of plant specimens were performed at the Herbarium of the Institute of Agricultural Research of Pernambuco (IPA-PE) (IPA n° 84037).

### Preparation of the Extracts

The dried bark (25 g) of *C. leptophloeos* was obtained by saturation in order of increasing polarity: submitted to Cyclohexane (CLCHE), Chloroform (CLCLE), Ethyl Acetate (CLAEE), Methanolic (CLMEE), and Aqueous (CLAQE) (250 mL) by agitation at 180 rotations per minute (rpm). After 24 h, the extract was filtered (Whatman^®^ number 2) and concentrated at 45°C under vacuum in a rotary evaporator (Concentrator 5301, Eppendorf^®^). The powder produced was kept at –20°C for future use. For phytochemical and antimicrobial analysis, the extracts were dissolved in your respective solvents at the concentration of 100 mg/mL for all biological assays.

### Phytochemical Analysis

#### Determination of Phenolic Acid Compounds by HPLC

For the determination of phenolic acids, the extract powder (0.5 g) was diluted in methanol: water (20%, v/v) at ultrasonic bath sonicator for 30 min. Then, the extracts were filtered and passed through a SPE C18 cartridge with the following solvents: acetone, trichloroacetic acid, water (4%, v/v) and methanol. Samples were later submitted to a rotary evaporator (Concentrator 5301, Eppendorf^®^) and re-suspended in methanol. The qualitative analysis of phenolic content for each extract was performed by UFLC (Ultra-Fast Liquid Chromatographic - LC-20AD, Shimadzu). Separations were conducted on a XR ODS, 50 μm × 3.0 μm × 2.2 μm column. The elution was performed with water: acetonitrile: methanol: ethyl acetate: glacial acetic acid (86:6:1:3:1, respectively). The column temperature was set to 40°C and the flow rate was 0.4 mL/minute for 5 min. Prior to injection, sample extracts (200 μL) were filtered with PTFE syringe 0.22 μm filters (Phenomenex, UK). Phenolics in each bark extract were identified by comparison of their retention times with corresponding standards and by their UV spectra obtained with the diode array detector – DAD (SPD-M20A). Gallic acid, vanillic acid, protocatechuic acid, chlorogenic acid, coumaric acid, ferulic acid, quercetin, and rutin were used as standard compounds (Prieto et al., 1999; Fernandes et al., 2011; Gómez-Caravaca et al., 2013). The linear regression equation for each standard curve was obtained by plotting the amount of standard compound injected against the peak area.

#### Qualitative Phytochemical Analysis by TLC

An aliquot of 100 μL of each *C. leptophloeos* extract was subjected to qualitative phytochemical analysis to ascertain the presence of secondary metabolites such as: coumarins (Gocan and Cimpan, 2007), flavonoids (Garcia et al., 1993), tannins and phytosteroids (Pascual et al., 2008), reducing sugars (Krishnamurthy et al., 2012), and saponins (Ng et al., 1994), respectively. The classes of compounds were visualized using Thin Layer Chromatography (TLC) on silica gel 60 F254 (Merck, Germany), and different systems of development and adequate visualization techniques were used as: Dragendorff test, NEU-PEG, KOH-Ethanol, Acetic Anhydride test, Vanillin-sulfuric acid, Quercetin, Tannic acid, Benzopyrone equivalent, according to the respective method of elucidation.

#### Determination of Total Phenol Content

The total amount of phenolic compounds from the extracts was determined according to the Folin-Ciocalteu procedure with *in house* modifications (Singleton et al., 1965). Briefly, samples (200 μL) were introduced into test tubes with 1.0 mL of Folin–Ciocalteu reagent (1:1 v/v) and 2.5 mL of sodium carbonate (20%). The mixture was incubated for 30 min at room temperature and allowed to stand still for additional 30 min. The absorbance from the blue colored mixture was measured at 765 nm (Gene Quant 1300, GE Healthcare). The amount of total phenol was calculated as milligrams (mg) of Gallic Acid Equivalents (GAE)/g of dry mass from calibration curve of Gallic acid standard solution. For the Gallic acid, the curve absorbance *versus* concentration is described by the equation *y* = 1.5221x + 0.0081 (*r*^2^ = 0.9712).

#### Chromatographic Analysis by Flash and CCD Chromatography

Based on the initial phytochemical results and visualization of the presence of phytosteroids and lignans in qualitative tests, the 0.8 g yield CLCLE was then submitted to Flash Chromatography (Clark et al., 1978; Still, 2002) using silica gel 60 F254 (Merck, Germany) as the stationary phase and CHCl_3_/MeoH (99.1) as the mobile phase. From this experiment, 45 fractions with 15 mL each were obtained. The fractions were submitted to Thin Layer Chromatography (TLC) in different groups depending on their chromatography patterns and similarity as follows: F1-F8, F9, F10-F14, F15-19, F20-26, F27-F28, F29-F38, F39-F42 e F44-F45. The fraction F9, which yielded 0.06 g was once submitted to silica gel column chromatography and 100 new fractions were obtained and analyzed following the data from Cambridge Crystallographic Data Centre (CCDC). Among all fractions, F16 was the purest one and was the submitted to ^1^H RMN.

#### Nuclear Magnetic Resonance (^1^H-NMR) Spectroscopy

The ^1^H NMR spectra of F16 fraction were obtained in Mercury-Varian spectrometer using 200 MHz – ^1^H. The solvent used was deuterated chloroform (CDCl_3_) whose respective peaks in RMN ^1^H were used to adjust the frequency scale.

### Antimicrobial Activities

#### Microorganisms and Inocula Preparation

Fifteen microorganisms strains from eleven species (*Aspergillus sp*., *Bacillus subtilis*, *Candida albicans*, *Enterococcus faecalis*, *Escherichia coli*, *Klebsiella pneumoniae*, *Micrococcus luteus*, *Mycobacterium smegmatis*, *Mycobacterium tuberculosis*, *Pseudomonas aeruginosa*, including clinical isolates of *Staphylococcus aureus*), obtained from the microorganism collection of the Department of Antibiotics of Federal University of Pernambuco (UFPEDA), were used for the antimicrobial tests, according to **Table 1**. The bacterial strains were cultured at 37°C for 18 hours in Mueller-Hinton Broth and the fungal cultures maintained in Sabouraud Dextrose Agar medium and incubated at 35°C for 24 h. As to the preparation of the inoculum, the pricked out strains were transferred to the sterile saline solution (0.9% NaCl), composed of a bacterial/fungal suspension (inoculum) until obtaining the concentration of 10^6^UFC/mL according to the scale of McFarland. The susceptibility tests were performed by Mueller Hinton agar-well diffusion method (Davis and Stout, 1971; Hombach et al., 2013).

**Table 1:** Microorganisms used in the present study distributed according to groups: bacteria and fungi

Bacteria	UFPEDA	Bacteria	UFPEDA	Fungi	UFPEDA
*Mycobacterium smegmatis*	71	*Staphylococcus aureus*	02	*Aspergillus* sp.	807
*Mycobacterium tuberculosis*	82	*Staphylococcus aureus*	672^1^	*Candida albicans*	1007
*Bacillus subtilis*	86	*Staphylococcus aureus*	677^1^		
*Micrococcus luteus*	100	*Staphylococcus aureus*	682^1^		
*Enterococcus faecalis*	138	*Staphylococcus aureus*	728^1^		
*Escherichia coli*	224				
*Klebsiella pneumoniae*	396				
*Pseudomonas aeruginosa*	416				


*ATCC, American Type Culture Collection. ^1^Infection site of *Staphylococcus aureus* strains; 672, blood; 677, postoperative secretion; 682, ocular discharge; 728, oropharynx.*

#### Minimum Inhibitory Concentration (MIC) and Minimal Microbicidal Concentration (MMC) Tests

The MIC and MMC were determined for plant extracts that showed antimicrobial activity, by a broth microdilution method (Weinstein et al., 2012; Hombach et al., 2013). Briefly, 100 μL of Mueller–Hinton Broth plus different concentrations of plant extracts were prepared and transferred to each microplate well to obtain dilutions of the active extract, ranging from 0.001 to 100 mg/mL. Then, 10 μL of a fresh culture (final concentration of 1 × 10^6^CFU/mL) of test organisms was added. Microplates were incubated at 37°C for 24 h and MIC was defined as the lowest concentration of the extract that restricted the visible growth of microorganism tested. To determine MMC, 100 μL from each well that showed no visible growth was reinoculated on MH agar plates; then the plates were incubated at 37°C for 24 h. MMC was defined as the lowest extract concentration showing no bacterial growth, DMSO was used as blank.

#### Antimycobacterial Culture

The *M. smegmatis* and *M. tuberculosis* was maintained on Middlebrook 7H9 broth containing 0.05% Tween 80 and 10% (v/v) OADC (Oleic Acid, Albumin, Dextrose, and Catalase) supplement. The culture screening was performed by Ziehl–Neelsen staining before used in the antimicrobial assays. Two-fold serial dilutions of CLCLE extract and Rifampicin were made with 100 μl of each; sterile distilled water and 7H9 Middlebrook culture medium for *M. tuberculosis* and *M. smegmatis* in plates of 96 well microplates. The plates were incubated at 37°C for 24 days. The developer (40 μL) used was iodonitrotetrazolium (INT) of Sigma–Aldrich. Minimum Inhibitory Concentration (MIC) values were recorded as the lowest concentrations of extracts showing no growth, and bacterial growth in the wells was indicated by color change (Damato and Collins, 1990; Sadaphal et al., 2008).

#### Antimicrobial Activity of *C. leptophloeos* Hinokinin against Selected MRSA Clinical Isolates

The study included four *S. aureus* strains obtained from the Department of Antibiotics of Federal University of Pernambuco (UFPEDA). Colonies with macroscopic characteristics of antimicrobial susceptibility patterns from isolates were determined according to Kirby Bauer disk diffusion technique as described by CLSI (Hombach et al., 2013) and Minimum Inhibitory Concentration (MIC). The following three antibiotics were used to determine the antibiogram of the isolates: Tobramycin (10 μg), Vancomycin (30 μg), and Cefoxitin (30 μg). Detection of methicillin resistant *S. aureus* (MRSA) was carried out using cefoxitin (30 μg), an inhibition zone diameter of ≤21 mm was reported as methicillin/oxacillin resistant and ≥22 mm was considered as methicillin/oxacillin sensitive (Hombach et al., 2013; CLSI, 2014; Rabelo et al., 2014).

### *In vitro* Hemolytic Assays

For hemolytic *in vitro* assay, whole blood (5 ml) was obtained from healthy, non-smoking volunteers by venipuncture, after obtaining written informed consent (National Ethics Committee reference number 30667014.5.0000.5208). Human erythrocytes from citrated blood were immediately isolated by centrifugation at 1500 rpm for 10 min at 4°C. After plasma removal, the erythrocytes were washed three times with phosphate-buffered saline (PBS; pH 7.4) and then re-suspended using the same buffer, and a 1% erythrocyte suspension was prepared. The hemolytic activity of *C. leptophloeos* extracts was tested under *in vitro* conditions. Each tube received 1.1 mL of erythrocyte suspension and 0.4 mL of extract with different concentrations ranging from 50 to 500 μg/mL. The negative control was solvent only and the positive control received 0.4 mL of Quillaja saponin (0.0025%). After 60 min of incubation at room temperature, cells were centrifuged at 1500 rpm for 10 min and the supernatant was used to measure the absorbance of the liberated hemoglobin at 540 nm length. The average value was calculated from triplicate assays (Sulaiman and Gopalakrishnan, 2013).

### Statistical Analysis

Each experiment was performed in biological duplicates and technical triplicates and results are presented as means and ± standard deviation (SD). Statistical analysis was performed by Student’s *t*-test and ANOVA tests. Differences were considered significant at *p* < 0.05. The concentration needed for 50% inhibition (IC_50_) was estimated graphically by linear regression analysis.

## Results

### Phytochemical Analyses of *C. leptophloeos*

**FIGURE 1:**
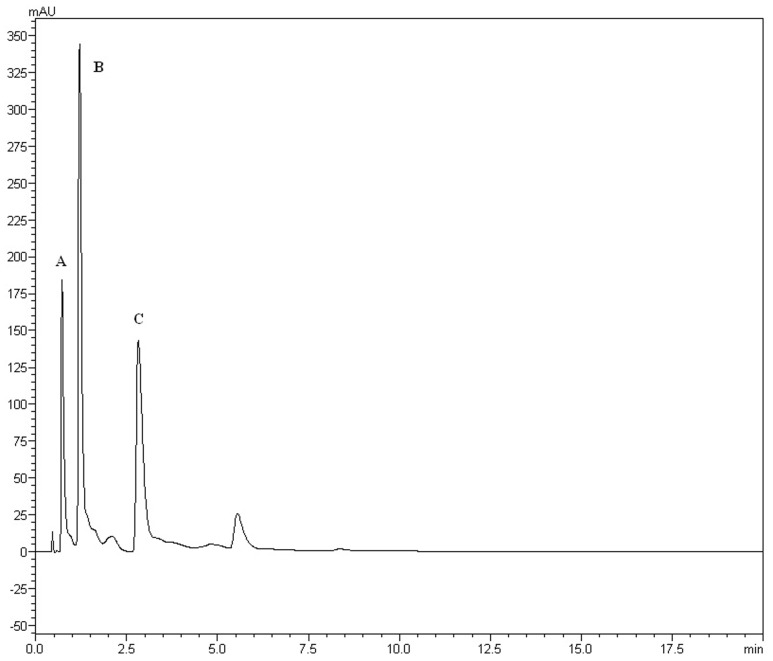
**HPLC fingerprints obtained by Chloroform extract (CLECL).** The UFLC analysis detected the presence of the bioactive compounds: **(A)** Gallic acid, **(B)** Chlorogenic acid, and **(C)** Protocatechuic acid.

**FIGURE 2:**
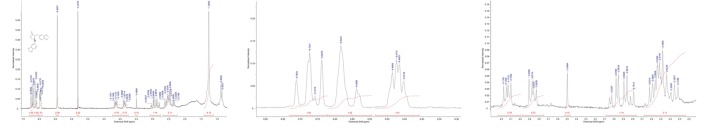
**^1^H NMR spectra of F16 fraction (δ, CDCl_3_, 200 MHz) of Hinokinin 2D structure by ACD/I-Lab**.

The yields of the assessed extracts presented values varying between 9.8 and 12.3%, being 9.8% for CLCHE, 10.2% for CLECL, 10.5% for CLAEE, 11.8% for CLMEE, and 12.3% for CLAQE. The qualitative phytochemical analysis of *C. leptophloeos* by HPLC detected the presence of three particular compounds: Gallic acid (GA), Chlorogenic acid (CGA), and Protocatechuic acid (PCA), as showed in **Figure 1**. The qualitative phytochemical analysis by TLC of *C. leptophloeos extracts* detected the presence of Phenolic compounds, Flavonoids and Reducing sugars in all extracts (**Table 2**). The estimation of total phenolic content revealed that CLAQE (33.64 ± 0.5 mg of GAE/g) and CLMEE (20.3 ± 0.78 mg of GAE/g) exhibited the highest phenolic content (*p* < 0.05). The other extracts showed phenolic content values ranging from 13.8 ± 0.53 to 12.54 ± 0.55 mg of GAE/g, showed in **Table 2**. The fraction F16 from CLCLE studied shown the presence of hinokinin in our analysis and the spectrum ^1^H NMR (CDCl_3_, 200 MHz) of F16 from CLCLE showed two multiplets (δH 2.45 e 2.48) in high field, being referent to sp^3^ hydrogens connected to neighboring carbons of chiral carbon (C-7 and C-7’). In δH 2.85 was observed a doublet of doublet (dd, *J* = 4.7, 14.5 Hz,1H) of 1 H connected directly to chiral carbon C-8. In δH 3.00 the doublet of doublet (dd, *J* = 4.7, 14.5 Hz,1H) of hydrogen 1 connected directly to the chiral carbon (C-8’). In δH 3.85 (dd, *J* = 7.0, 9.2 Hz,1H) and 4.15 (dd, *J* = 6.2, 9.0 Hz,1H) two doublets of doublets regarding the hydrogens linked to the carbon C-9. In δH 5.9471 a multiplet in 4 H linked to the carbon C-10 and C-10’, simultaneously. Finally, a 6H a multiplet in δH 6.5 assigned to aromatic hydrogens were identified. The spectrum elucidation is show in **Figure 2**. Hinokinin is one of the constituents of secondary metabolites of *C. leptophloeos* described for the first time in this species.

**Table 2:** Phytochemical analyses of extracts from barks of *Commiphora leptophloeos*.

*Commiphora leptophloeos* Extract	Phenolic contents	Phytochemical screen	
	
		Positive tests for	Negative tests for
CLAQE	33.64 ± 0.5	Phenolic compounds, Tannins, Flavonoids, and Reducing sugars.	Coumarins, Saponin, Phytosteroids, and Lignans.
CLMEE	20.3 ± 0.78	Phenolic compounds, Tannins, Coumarins, Flavonoids, Reducing sugars, and Saponin.	Phytosteroids and Lignans.
CLCLE	12.54 ± 0.55	Phenolic compounds, Tannins, Coumarins, Flavonoids, Reducing sugars, Phytosteroids, and Lignans.	Saponin
CLCHE	13.8 ± 0.53	Phenolic compounds, Tannins, Coumarins, Flavonoids, and Reducing sugars.	Saponin, Phytosteroids, and Lignans.
CLAEE	13.7 ± 0.04	Phenolic compounds, Flavonoids, and Reducing sugars.	Tannins, Coumarins, Saponin, Phytosteroids, and Lignans.


*CLAQE, *C. leptophloeos* aqueous extract; CLMEE, Methanolic extract; CLCLE, Cloroform extract; CLCHE, Cyclohexane extract and CLAEE, Ethyl acetate extract. Phenolic contents are expressed in mg/g of Gallic Acid Equivalent (GAE). Data were obtained from three independent experiments, each performed in triplicates (*n* = 3) and represented as mean ± SD and *p*-value < 0.05.*

### Antimicrobial Screening

The antibacterial activity of *C. leptophloeos* extracts was recorded against various microorganisms and is presented in **Tables 3**–**5**. Overall, all plant extracts exhibit a range of inhibitory potentials with broad spectrum, as they inhibited all bacteria and yeasts species tested. The better antimicrobial results observed were provided by CLMEE, in which MIC ranged from 0.097 to 50.0 mg/mL (*p*-value < 0.05) (**Tables 3** and **4**). CLMEE presented better antimicrobial activities against Gram-positive bacteria with best results for *B. subtilis* (MIC = 3.125 mg/mL), *E. faecalis* (MIC = 25 mg/mL) and *M. luteus* (MIC = 0.097 mg/mL and MMC = 12.5 mg/mL). Against *S. aureus* strain, the best bacteriostatic action was the CLCLE (MIC = 1.125 mg/mL) and, for this reason, we use F16 fraction purified *C. leptophloeos* hinokinin to evaluate the action of this lignan against MRSA strains (**Table 5**). *C. leptophloeos* also showed antifungal activity, such as *Aspergillus sp.* (MIC values ranged from 3.125 to 6.25 mg/mL) and *C. albicans* (MIC values ranged from 6.25 to 12.5 mg/mL, *p*-value < 0.05) showed in **Table 3**. The antimicrobial activities against Gram-negative bacteria were showed in **Table 4**. CLCHE showed better bacteriostatic effect against *P. aeruginosa* (MIC = 6.25 mg/mL) and *K. pneumoniae* (MIC and MMC = MMC 12.5 mg/mL), CLAEE showed the better activity against *E. coli* (MIC and MMC = 12.5 mg/mL), and CLCLE was tested against *Mycobacterium*, indicating *M. smegmatis* (MIC = 12.5 mg/mL) to be more susceptible to CLCLE than *M. tuberculosis* (MIC = 52 mg/mL), **Table 4**. The MIC values of hinokinin (obtained from the purification F16 from CLCLE) ranged from 0.0485 to 3.125 mg/mL (*p*-value = 0.002) for the different *S. aureus* clinical isolates tested, and showed a bactericidal activity against MRSA isolated from blood (MMC 0.40 mg/mL) and postoperative secretion (MMC = 3.125 mg/mL) showed in **Table 5**.

**Table 3:** Antimicrobial activity of extracts from barks of *C. leptophloeos* against selected Gram-positive bacteria and fungi.

*Commiphora leptophloeos Extract*	*Bacillus subtilis*		*Enterococcus faecalis*		*Micrococcus luteus*		*Staphylococcus aureus*		*Aspergillus sp.*		*Candida albicans*	
						
	MIC	MMC	MIC	MMC	MIC	MMC	MIC	MMC	MIC	MMC	MIC	MMC
CLMEE	3.125	–	25	–	0.097	12.5	1.56	–	3.15	–	6.25	–
CLCLE	25.0	–	25	–	0.78	25	1.125	–	6.25	–	12.5	–
CLCHE	12.5	–	25	–	0.195	–	3.125	–	6.25	–	6.25	–
CLAEE	12.5	–	25	–	12.5	12.5	12.5	12.5	3.125	–	12.5	–

**Control**	**MIC**		**MIC**		**MIC**		**MIC**		**MIC**		**MIC**	

KAN	4		0.614		6.8		1.6		N.d.		3.2	
KCZ	N.d.		N.d.		N.d.		N.d.		0.32		N.d.	


*MIC, Minimal Inhibitory Concentration; MMC, Minimal Microbicidal Concentration; MIC and MMC values are expressed in mg/mL. CLAQE, C. *leptophloeos* aqueous extract; CLMEE, Methanolic extract; CLCLE, Cloroform extract; CLCHE, Cyclohexane extract and CLAEE, Ethyl acetate extract. KAN, Kanamycin and KCZ, Ketoconazole. N.d., Not determined. Data were obtained from three independent experiments, each performed in triplicates (*n* = 3) and represented as mean ± SD and *p*-value < 0.05.*

**Table 4:** Antimicrobial activity of extracts from barks of *C. leptophloeos* against selected Gram-negative bacteria and Mycobacterium.

*Commiphora leptophloeos Extract*	*Pseudomonas aeruginosa*		*Escherichia coli*		*Klebsiella pneumoniae*		*Mycobacterium smegmatis*		*Mycobacterium tuberculosis*	
						
	MIC	MMC	MIC	MMC	MIC	MMC	MIC	MMC	MIC	MMC
CLMEE	50.0	–	12.5	25	12.5	–	N.d.	N.d.	N.d.	N.d.
CLCLE	50.0	–	12.5	–	12.5	–	12.5	–	54.1	–
CLCHE	6.25	–	12.5	–	12.5	12.5	N.d.	N.d.	N.d.	N.d.
CLAEE	–	–	12.5	12.5	12.5	25	N.d.	N.d.	N.d.	N.d.

**Control**	**MIC**		**MIC**		**MIC**		**MIC**		**MIC**	

AMP	0.008		0.004		0.008		N.d.		N.d.	
RIF	N.d.		N.d.		N.d.		1.16		2.5	


*MIC, Minimal Inhibitory Concentration; MMC, Minimal Microbicidal Concentration; MIC and MMC values are expressed in mg/mL. CLAQE, C. *leptophloeos* aqueous extract; CLMEE, Methanolic extract; CLCLE, Cloroform extract; CLCHE, Cyclohexane extract and CLAEE, Ethyl acetate extract. AMP, Ampicillin and RIF, Rifampicin. N.d., Not determined. Data were obtained from three independent experiments, each performed in triplicates (*n* = 3) and represented as mean ± SD and *p*-value < 0.05.*

**Table 5:** Antimicrobial Activity of *C. leptophloeos* hinokinin against selected *S. aureus* clinical isolates.

UFPEDA	Source^1^	HKN		VA	TOB	CFX
			
		MIC	MMC	IDZ		
672	Blood	0.395	0.400	11.4	13.9	14.2
677	Postoperative secretion	0.0485	3.125	15.1	16	14.2
682	Ocular discharge	3.125	–	13.3	12	15
728	Oropharynx	1.560	–	14	14.8	16


*MIC, Minimal Inhibitory Concentration; MMC, Minimal Microbicidal Concentration; MIC and MMC values are expressed in mg/mL. IDZ Inhibition Disc Zone are expressed in mm according CLSI. HK, Hinokinin; VA, Vancomycin; TOB, Tobramycin and CFX, Cefoxitin. ^1^Source of *S. aureus* strains; 682, ocular discharge; 672, blood; 677, postoperative secretion; 728, oropharynx. Data were obtained from three independent experiments, each performed in triplicates (*n* = 3) and represented as mean ± SD and *p*-value < 0.05.*

### *In vitro* Hemolytic Assays

The concentration from CLAQE, CLMEE, CLAEE, CLCLEE, and CLCHE extracts demonstrated a HC_50_ (concentration required for 50% of hemolysis) of 313 ± 0.5 μg/mL; 304.9 μg/mL ± 0.8; 287.49 μg/mL ± 3.0; 239.5 μg/mL ± 1.4, and 177.21 μg/mL ± 0.45, respectively (*p*-value = 0.001).

## Discussion

### Phytochemical Analyses of *C. leptophloeos*

Species from *Commiphora* genus present bioactive compounds widely known and used as therapy for several pathologies in folk culture. Herein, we performed a thorough phytochemical characterization of *C. leptophloeos*, showing its notable amounts of phenolic compounds, namely Gallic acid (GA), Chlorogenic acid (CGA), and Protocatechuic acid (PCA). GA is endowed with pharmacological activities, including antioxidant, anti-inflammatory, antimicrobial and antiproliferative activity (Kroes et al., 1992; Vishnu Prasad et al., 2010). On the other hand, CGA has been recently pointed as modulator of glucose and lipid metabolism *in vivo*, upon unbalanced metabolic conditions such as diabetes (Lou et al., 2011; Sato et al., 2011; Zhao et al., 2012; Hwang et al., 2014). Additionally, PCA, the major metabolite of anthocyanin, provides beneficial activities to human’s health such as reduced risk of cardiovascular diseases (Wang et al., 2010), anti-inflammatory, antioxidant and free radical scavenging activities (Li, 2011), as well as estrogenic and antiestrogenic activity (Kakkar and Bais, 2014).

Our phytochemical characterization showed the presence of other bioactive compounds in *C. leptophloeos*, such as hinokinin, an important class of lignans, which has been recently investigated in order to establish its biological activities (REFERENCIA). Lignans are important components of food and medicines biosynthetically deriving from the radical coupling of two molecules of coniferyl alcohol at C-8/C-8′ positions. In the past years, the biological activities of several lignans have been studied in depth (Aehle et al., 2011; Zhang et al., 2014) and among them, the hinokinin (**Figure 2**) is emerging as a new interesting compound with pharmacological potential. Hinokinin was isolated for the first time in 1933 by Yoshiki and Ishiguro from an ether extract of *Hinoki* wood – *Chamaecyparis obtuse* - as a colorless crystalline compound and over the years it has been gradually characterized and described by other researchers (Vanoeveren et al., 1994; Timple et al., 2013; Desai et al., 2014).

### Antimicrobial Screening

*Commiphora leptophloeos* shows antibacterial activity against several human Gram-positive pathogens such as *B. subtilis*, *E. faecalis*, *M. luteus*, and *S. aureus* (**Table 3**). The inhibition of bacterial growth *in vitro* by the extracts of *C. leptophloeos* could be due to the presence of some active compounds including flavonoids, phenolic acids and tannins, described in **Table 2**, known to be effective antimicrobial agents against a wide array of microorganisms. These active compounds may act alone or in combination to inhibit bacterial growth. Our results against *M. luteus* strain showed a MIC ranging from 0.097 to 12.5 mg/mL, and the CLMEE as the extract showing the best bacteriostatic and bactericidal action (MMC = 12.5 mg/mL), particularly when compared to other studies with Commiphora genus (Latha et al., 2005) and when compared with the MIC value obtained by the aminoglycoside Kanamycin (MIC = 6.8 mg/mL). *M. luteus* is a natural constituent of mammalian skin microflora and considered a nosocomial contaminant mainly in immunodeficient individuals, causing meningitis (Fosse et al., 1985) and endocarditis (Miltiadous and Elisaf, 2011) in severe infections. Immunodeficiency is a risk factor able to intensify selection and dissemination of multidrug resistance strains. *C. leptophloeos* extracts are also effective against *Aspergillus sp*. and *C. albicans*, the most prevalent fungal species of the human microbiota, often associated to several complications in immunosuppressed individuals (Nobile and Johnson, 2015).

Gram-negative pathogens are particularly alarming, due to their resistance to nearly all drugs firstly considered for treatment. The reason for this relies on their several pathways to β-lactam resistance through β-lactamases enzymes production, therefore interfering with the mechanism of action of β-lactam antibiotics. The same premise has become more frequent in Gram-positive infections (e.g., Staphylococcus). *P. aeruginosa* is a ubiquitous opportunistic pathogen, having outer membrane cell structure conferring pronounced resistance to xenobiotics (Gaspar et al., 2013). Previous studies had already reported the action of the aqueous extract of *C. leptophloeos* inhibition of biofilms of *P. aeruginosa* (Trentin et al., 2013) and *S. epidermidis* (Trentin et al., 2011). According to these aforementioned studies, CLCHE presented ample bacteriostatic activity against *P. aeruginosa*, with MIC of 6.25 mg/mL (**Table 4**), this greater potential of action is due the presence of tannins in CLCHE, as noted in our phytochemical profile (**Table 2**). *E. coli* is a bacterium usually found in intestinal microflora, nevertheless, some can cause debilitating and sometimes fatal human diseases (Riley, 2014). Pathogenic strains are divided into intestinal pathogens causing diarrhea and extraintestinal mainly related to urinary tract infections pyelonephritis, cystitis, and urosepsis (Croxen et al., 2013). Only two fractions, CLCHE and the CLMEE, showed bactericidal action (MMC ranging 12.5 to 25 mg/mL), suggesting that this action may be due to the presence of high amounts of phenolic acids (**Figure 1**), such as GA, which might contribute to the inhibition *E. coli* strain.

*Klebsiella pneumoniae* is a type of Gram-negative bacteria that may cause different types of infections including pneumonia, meningitis, bloodstream infections. Generation of extended- spectrum β- lactamases is one of the major mechanisms by which clinical *K. pneumoniae* develop resistance to antibiotics (Cai et al., 2016). Herein we identified that the CLCHE and CLAEE fractions effectively act as antibacterial agent in the Gram-negative tested strains (**Table 4**). The antibacterial activities of *C. leptophloeos* extracts were also detected against *M. smegmatis* and *M. tuberculosis* strains as showed in **Table 4**. A bacteriostatic effect against Mycobacterium species by plants secondary metabolites may be due to pathogen’s thick outer membrane that is highly hydrophobic and possibly provided a permeability to the extract (Yamori et al., 1992; Firmani and Riley, 2002).

Oxacillin and methicillin resistant *S. aureus* (MRSA) are resistant to all β-lactam agents including cephalosporin’s and carbapenems, causing global commitment in stopping its spread responsible for approximately 40% of *S. aureus* infections in global Intensive Care Units (ICU) (Frieden, 2013; Cuny et al., 2015). In our study, were evaluated four MRSA clinical isolated strains reported as Methicillin Resistance (Oxacillin Resistance) confirmed with CFX inhibition disc tests at where cefoxitin is used as a surrogate for mecA-mediated oxacillin resistance (CLSI, 2014). *C. leptophloeos* hinokinin showed the highest antibacterial activity against MRSA isolated of blood, with a bacteriostatic activity (MIC) of 0.39 mg/mL and bactericidal (MMC) of 0.40 mg/mL, both with very similar values (**Table 5**). Our results point towards a promising antimicrobial potential against *S. aureus* resistant, especially when compared to others studies (Saeed and Sabir, 2004; Abdallah et al., 2009; Abdulgader et al., 2015), once we obtained more pronounced antibacterial results, corroborating the antimicrobial potential of hinokinin.

Regarding hemolytic activity of *C. leptophloeos* extracts, the values obtained for hemolysis were superior to the ones regarding its antimicrobial activity, showing its safety. Even though a phytochemical study on another Commiphora genus (Hanus et al., 2005) has shown the presence of possibly toxic compounds, the toxicity assay performed *in vitro* for Amburana hemolytic properties did not show the same results. Regardless, we recognize the importance of applying other methods to assess toxicity from these extracts over other cellular components. Hemolytic assays were performed to assess cell safety in future pharmacological preparations without causing any harm. In the present study our trials with Commiphora showed lower hemolytic activity when comparing to other species from the Caatinga biome (Silva et al., 2011; Tam et al., 2015).

## Conclusion

In the present study, we identified that the bark extracts from *C. leptophloeos* contain an important amount of phenolic compounds, such as GA, PCA, and CGA. Furthermore, we have identified for the first time the presence of hinokinin in Commiphora genus. Our results showed that *C. leptophloeos* presents potential inhibitory properties against *S. aureus* multi-drugs resistance species, as well as several Gram-positives, Gram-negatives and fungi, and should also be studied for their potential against *Mycobacterium*.

## Author Contributions

JdS, performed plant collection, biochemical and antimicrobial assays, analysis and wrote the paper; AdP, performed plant collection and participated in all biochemical and microorganism’s assays; JJ, performed the collection and all microorganisms’ assays; JdP, performed chemical assays and analysis; SC, discussed the results and helped in its delimitation from for the final manuscript; MdS, Coordinated the project, participated in plant collection, during all biochemical assays, chemical analysis, and in manuscript writing; JdA, Coordinated the project including all plant collection, biochemical, chemical, and biological assays and discussed all results.

## Conflict of Interest Statement

The authors declare that the research was conducted in the absence of any commercial or financial relationships that could be construed as a potential conflict of interest.
